# Genomic Analysis of Prophages Recovered from *Listeria monocytogenes* Lysogens Found in Seafood and Seafood-Related Environment

**DOI:** 10.3390/microorganisms9071354

**Published:** 2021-06-22

**Authors:** Hue Thi Kim Vu, Matthew J. Stasiewicz, Soottawat Benjakul, Kitiya Vongkamjan

**Affiliations:** 1 Department of Food Technology, Prince of Songkla University, Hat Yai, Songkhla 90112, Thailand; kimhue300887@gmail.com; 2 Department of Veterinary Hygiene and Food Safety, National Institute of Veterinary Research (NIVR), Dong Da, Hanoi 11519, Vietnam; 3 Department of Food Science and Human Nutrition, University of Illinois at Urbana-Champaign, Urbana, IL 61801, USA; mstasie@illinois.edu; 4 International Center of Excellence in Seafood Sciences, Faculty of Agro-Industry, Prince of Songkla University, Hat Yai, Songkhla 90112, Thailand; soottawat.b@psu.ac.th; 5 Department of Biotechnology, Faculty of Agro-Industry, Kasetsart University, Chatuchak, Bangkok 10900, Thailand

**Keywords:** comparative genomic analysis, *L. monocytogenes* lysogen, *Listeria* prophage, phage sequencing analysis

## Abstract

A prophage is a phage-related sequence that is integrated into a bacterial chromosome. Prophages play an important role in bacterial evolution, survival, and persistence. To understand the impact of *Listeria* prophages on their host genome organizations, this work sequenced two *L. monocytogenes* strains (134LM and 036LM), previously identified as lysogens by mitomycin C induction. Draft genomes were generated with assembly sizes of 2,953,877 bp and 3,000,399 bp. One intact prophage (39,532 bp) was inserted into the *comK* gene of the 134LM genome. Two intact prophages (48,684 bp and 39,488 bp) were inserted in tRNA-Lys and elongation-factor genes of the 036LM genome. The findings confirmed the presence of three corresponding induced phages previously obtained by mitomycin C induction. Comparative genomic analysis of three prophages obtained in the newly sequenced lysogens with 61 prophages found in *L. monocytogenes* genomes, available in public databases, identified six major clusters using whole genome-based phylogenetic analysis. The results of the comparative genomic analysis of the prophage sequences provides knowledge about the diversity of *Listeria* prophages and their distribution among *Listeria* genomes in diverse environments, including different sources or geographical regions. In addition, the prophage sequences and their insertion sites contribute to the genomic diversity of *L. monocytogenes* genomes. These data of prophage sequences, prophage insertion sites, and prophage sequence comparisons, together with ANIb confirmation, could be useful for *L. monocytogenes* classification by prophages. One potential development could be refinement of prophage typing tools for monitoring or surveillance of *L. monocytogenes* contamination and transmission.

## 1. Introduction

*L*. *monocytogenes* is a foodborne pathogen and the causative agent of listeriosis, a potentially fatal infection with a mortality rate up to 30% [1,2]. A number of listeriosis outbreaks have been linked to contamination of *L. monocytogenes* in a variety of foods [3,4], including seafood. *L. monocytogenes* is well adapted to survive in stressful environments [5,6,7], which enhance persistence in food associated environments [8].

Comparative genomic analysis of *L. monocytogenes* isolates from food environments previously showed that prophages play an important role in the evolution of *L. monocytogenes* [9], facilitating the survival and persistence of this pathogen over time [10,11,12]. Prophages could contribute to the genetic variation of *Listeria* lysogens (prophage-carrying *Listeria*) [13,14]. They are commonly found in the genomes of *L. monocytogenes.* Multiple prophages, or monocins (an incomplete phage particle derived from a cryptic prophage), were observed in multiple strains, such as F6854, L99, HCC23, J0161 [9,13]. In addition, previous studies have also shown that prophages could serve as epidemiological markers for *L. monocytogenes* [15]. Therefore, the study of prophage diversity contributes to understanding bacterial genetic variation [16].

Several temperate *Listeria* phages have been sequenced [17,18,19]. They showed a basic genome organization comprising of: (i) DNA packaging; (ii) head structural component and assembly; (iii) tail and base plate; (iv) cell lysis; (v) lysogeny control; and (vi) DNA replication, recombination, modification, and gene expression. Most previously sequenced temperate phages were isolated from various natural environments, including phages LP-030-2 and LP-101 from dairy farm samples [20], and phages vB_LmoS_188 and vB_LmoS_293 from mushroom and mushroom compost [21]. In addition, genomes of three *Listeria* phages induced from the lysogens found in food and food-related environment were sequenced in a previous study [22]. The sequences of these induced phages were carried out in this study to compare with sequences of other known temperate *Listeria* phages.

Specifically, genomes of two selected *L. monocytogenes* lysogens isolated from seafood and a seafood-related environment were sequenced to explore the prophage regions or any potential reorganization, compared to the genome sequences of corresponding induced phages. Potential insertion sites and attachment sites were predicted to confirm the prophage border in the host *Listeria* genomes. Analysis of the prophage sequences was performed with sequences of their corresponding induced phages and those publicly available. Overall, information obtained here allows us to better understand (i) the relationship between prophages and prophage-related regions in the genomes of *L. monocytogenes* and (ii) the genomic diversity of temperate *Listeria* phages from different environments or various geographical regions.

## 2. Materials and Methods

### 2.1. Selection of Representative L. monocytogenes Lysogens for Genome Sequencing

To understand the prophage content and their organization in the *L. monocytogenes* lysogens, 2 isolates (PSU-KV-134LM and PSU-KV-036LM, hereafter identified as 134LM and 036LM, respectively) were selected for sequencing. These two lysogens were isolated from different sources, including a seafood product (fish stick) by Vongkamjan et al. (2016) [23] and a seafood processing environment by Vongkamjan et al. (2015) [24]. The *L. monocytogenes* lysogen 134LM harbors one inducible prophage and the *L. monocytogenes* lysogen 036LM harbors two inducible prophages [25].

### 2.2. DNA Extraction and Library Preparation for Sequencing

A single colony of each *Listeria* isolate was inoculated in 10 mL of Brain Heart Infusion (BHI, Oxoid, UK) broth and incubated at 30 °C with shaking (220 rpm) for 16 h, followed by a centrifugation at 6000 rpm for one minute before cell harvesting. Then, bacterial DNA was extracted using GF-1 bacterial DNA extraction kit (Vivantis, Selangor Darul Ehsan, Malaysia) following the manufacturer’s instruction. DNA quality and concentration were measured with a Maestro Nano Drop Spectrophotometer (Green bioresearch, LA, USA). The extracted DNA was subsequently used for sequencing library preparation by random fragmentation of the DNA sample using Illumina TruSeq DNA PCR-free kit, followed by 5′ and 3′ adapter ligation. Then, the high-quality sequencing fragmentation libraries were sequenced using the Illumina HiSeq2500 platform with 100-bp paired-end reads at Macrogen Inc. (Seoul, Korea).

### 2.3. Genome Assembly and Annotation of L. monocytogenes Lysogens

After sequencing, raw reads was filtered to remove the low-quality reads using Trimmomatic [26], then de novo genome assembly was performed using SOAPdenovo2 [27]. Genome sequence of *L. monocytogenes* F2365 (GenBank accession no. NC-002973.6) was used as a reference genome for alignment and ordering contigs using MAUVE genome alignment version 2.4.0 [28]. Subsequently, automatic annotation was performed with the draft genomes using RAST [29]. Genomes were then submitted to the NCBI Prokaryotic Genomes Automatic Annotation Pipeline (PGAAP) (http://www.ncbi.nlm.nih.gov/genomes/static/Pipeline.html, accessed date 13 July 2018) before receiving the GenBank accession numbers. tRNAscan-SE [30] was used to predict tRNA genes. RNAmmer [31] was used to predict rRNA genes. In addition, genomes of the two *L. monocytogenes* lysogens were submitted to the Center for Genomic Epidemiology website (https://cge.cbs.dtu.dk/services/MLST/, accessed on 1 June 2021) MLST tool for MLST sequence typing [32].

For these two sequences, and any *L. monocytogenes* strains used below without the following information, the corresponding clonal complex (CC) and Lineage were defined in BIGSdb via links to the PubMLST website (https://pubmlst.org/data/profiles/lmonocytogenes.txt, accessed on 1 June 2021) [33,34]. Serotype group, ‘serogroup’, was determined based on the core genome MLST (cgMLST) allelic profiles [35]. Moreover, to evaluate the similarities between genomes of *L. monocytogenes* newly sequenced here and those previously sequenced, the average nucleotide identity based on BLAST+ (ANIb) was calculated using the JSpeciesWS webserver (http://jspecies.ribohost.com/jspeciesws/, accessed on 1 June 2021) [36].

### 2.4. Identification and Annotation of Prophages and Prophage-Related Regions in the Newly Sequenced Lysogenic L. monocytogenes Genomes

The draft genome of each *Listeria* isolate was submitted to PHASTER for identification of prophages and prophage-related regions [37]. This tool classifies the detected prophage regions as intact, questionable, or incomplete prophage, based on a total score obtained by the completeness of required genes to fully form an assembled virus. The “questionable” or “incomplete prophages” were reported as putatively defective prophage as lacks of many structural phage genes. Thus, these putatively defective prophages were exclude from our analysis. The intact prophage was confirmed by identifying the presence of the phage integrase gene (*int*) that was adjacent to a potential bacterial gene, which could be a potential insertion site. To identify a complete sequence of each identified intact prophage, full gene clusters of prophages [18] were considered together with the potential insertion site.

Each complete sequence of the intact prophage was extracted for comparative analysis starting from 3′ end of the corresponding phage integrase gene through the 5′ end of the last phage genes detected by PHASTER. Generally, this site was several genes after a phage endolysin gene. PHASTER also detects a prophage attachment (att) site by searching for a sequence region with short nucleotide repeats (12–80 nucleotides) in both left and right junction fragments (*attL* and *attR*).

Then, the extracted intact prophage sequence was submitted for annotation using RAST [29]. Annotation results from PHASTER [37] were also used. The annotations were curated and verified using BLAST [38] and Artemis [39]. Functional domains were predicted using InterPro (http://www.ebi.ac.uk/interpro, accessed on 1 June 2021) [40]. In addition, tRNA genes were predicted using tRNAscan-SE webserver [30].

### 2.5. Variation of Prophages Inserted in Multiple Loci of L. monocytogenes Genomes

A total of 163 *L. monocytogenes* strains with complete genomes available on the National Center for Biotechnology Information (NCBI) database were subjected to prophage screening by PHASTER (http://phaster.ca/, accessed on 1 June 2021) with default settings. “Intact prophage” were manually confirmed as the prophages by the presence of the attachment site (att), integrase gene, terminases, and structural viral proteins. All 163 *L. monocytogenes* strains were subjected to MLST sequence typing (ST) [32]. ST and prophage presence were used to select 35 *L. monocytogenes* strains for allow up analysis (Table 1). The 14 STs of selected *L. monocytogenes* strains have global distribution [41,42,43]. Further, the 10 *L. monocytogenes* of ST1 are of ST overrepresented in clinical and food isolates worldwide [44]. The information of *L. monocytogenes* sequence type (global distribution and represented in clinical and food isolates) and prophage complement (having complete prophage sequence(s) and identified attachment site(s)) were used in parallel for the selection of *L. monocytogenes* strains to be included in our study.

All of these detected intact prophages obtained from 35 *L. monocytogenes* genomes were submitted to PHASTER [37] for prophage prediction and confirmation. For each detected intact prophage, prophage insertion site and prophage borders were manually validated (as described above) and showed that containing prophage(s) with previously reported insertion sites, including two *comK*-prophages, ten tRNA-Lys prophages, tRNA-Arg, tRNA-Ser, tRNA-Thr, elongation factor gene, and/or ribosomal protein S9.

For comparative genomic analysis of prophages, the sequences of three obtained prophages in the two newly sequenced lysogens and this database set were included. Overall comparison of 64 prophage sequences was conducted using intergenomic distances inferred with the Genome-Blast Distance Phylogeny (GBDP) tool [45] using settings recommended for prokaryotic viruses. The intergenomic distances were used to infer a balanced minimum evolution tree with branch support from 100 pseudo-bootstrap replicates. A phylogenetic tree of the prophage sequence alignment was generated using VICTOR with default settings and visualized by FigTree v1.4.3 [46]. Linear genome visualizations of prophages within each identified cluster were generated using Easyfig 2.1 [47]. BLASTn was used to determine the nucleotide similarities of the prophage sequences found in two *L. monocytogenes* lysogens newly sequenced and the closely related prophages [38].

### 2.6. Comparison of Prophages Present in the Lysogenic Genomes and the Corresponding Induced Phages

Comparative genomic analysis was performed using sequences of the three prophages (pp-134LM-comK, pp-036LM-EF, and pp-036LM-tRNA-Lys) found in the genomes of the two *L. monocytogenes* lysogens sequenced in this study and three *Listeria* phages (LP019, LP040, and LP041) that were sequenced previously [22]. Induction by Mitomycin C have previously yielded one induced phage, LP019, from the isolate 134LM and two induced phages, LP040 and LP041, from the isolate 036LM [25]. Linear genome visualizations between each pair (prophage sequence and induced phage sequence) was conducted using Easyfig 2.1 [47]. The regions presenting differences between the prophage sequences and the induced *Listeria* phage sequences were extracted for nucleotide sequence alignment using Clustal Omega [48].

### 2.7. Comparative Genomic Analysis of the Newly Sequenced Listeria Phages and the Previously Sequenced Temperate Listeria Phages

Full genome sequences of all temperate *Listeria* phages available on the NCBI database were obtained (Table 2).

The whole genome comparison of these temperate phages and our newly sequenced phages was conducted using intergenomic distances inferred with the GBDP tool [45] under settings recommended for prokaryotic viruses. The intergenomic distances were used to infer a balanced minimum evolution tree with branch support from 100 pseudo-bootstrap replicates. A phylogenetic tree of the alignment was generated using VICTOR with default settings and visualized by FigTree v1.4.3 [46]. Nucleotide similarity of each newly sequenced *Listeria* phages and the closely related temperate *Listeria* phages was obtained by BLASTn [38]. Linear genome visualizations were generated using Easyfig 2.1 [47].

### 2.8. Nucleotide Sequence Accession Numbers

Sequences of *L. monocytogenes* lysogens 134M and 036LM were deposited at DDBJ/ENA/GenBank under the accession numbers QSWG00000000 and QOUY00000000, respectively. The versions described in this paper are QSWG01000000 and QOUY01000000. FASTQ raw sequence files for *L. monocytogenes* lysogens 134M and 036LM have been deposited in the Sequence Read Archive (SRA) database at NCBI with SRA accession number SRP162378 and SRP163030 under BioProject PRJNA484544 and PRJNA480937, respectively.

## 3. Results

### 3.1. Draft Genomes of Lysogenic L. monocytogenes

The draft genomes of *L. monocytogenes* 134LM and 036LM yielded 21 and 19 million reads with an estimated coverage of 715× and 643×, respectively (Table 3). Genome assembly size of *L. monocytogenes* 134LM was 2,953,877 bp with 2955 detected CDSs (11 contigs). Assembly size of *L. monocytogenes* 036LM was 3,000,399 bp with 3027 detected CDSs (11 contigs). The GC content of both *L. monocytogenes* lysogens was 37.8%. A total of 54 tRNAs and four complete rRNA operons (16S-23S-5S) were found in 134LM genome, while 52 tRNAs and three complete rRNA operons were found in 036LM genome. The genomes of these two lysogens showed 99.94% sequence similarity as observed by ANIb (Supplement Table S1), and shared 99.99% similarity to the genome of *L. monocytogenes* reference strain F2365. In addition, genomes of the two *L. monocytogenes* lysogens were classified into MLST sequence type 1 (CC1), lineage I, and belonged to *L. monocytogenes* serogroup IVb (which includes serotypes 4b, 4ab, 4d, and 4e).

### 3.2. Identification and Annotation of Prophages and Prophage-Related Regions in the Genomes of Lysogenic L. monocytogenes Newly Sequenced

**Figure 1:**
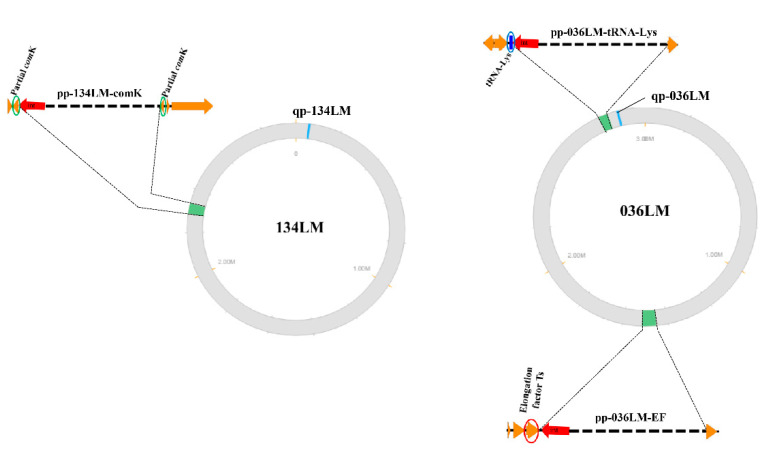
Locations of the intact prophages (pp) and questionable prophage (qp) detected in 134LM/036LM genomes.

PHASTER program identified one intact prophage in the 134LM genome (pp-134LM-comK; 39,532 bp) and two intact prophages in the 036LM genome (pp-036LM-EF; 48,684 bp and pp-036LM-tRNA-Lys; 39,488 bp) (Table 3 and Figure 1). In addition, a conserved questionable prophage (10,729 bp) was found in both genomes. The major differences between the two lysogenic genomes were the inserted prophage sequences: 90 kb inserted in the genome of 036LM and 40 kb inserted in the genome of 134LM. These prophages were found in different insertion sites. Those with a *comK*-prophage insertion resulted in a separation of the *comK* gene. However, prophage pp-036LM-EF was inserted in a translation elongation factor gene (EF) and prophage pp-036LM-tRNA-Lys was located at the 3′-end of tRNA-Lys of the 036LM genome. Various repeat nucleotide sequences as phage attachment sites were identified: (i) “ATGATTATAATAAT” for prophage pp-134LM-comK, (ii) “TGATAACAAAGC” for prophage pp-036LM-EF and (iii) “ACTCTTAATCAGCGGGTCGGGGGTTCGAAACCCTCACAACCCATA” for prophage pp-036LM-tRNA-Lys.

**Figure 2:**
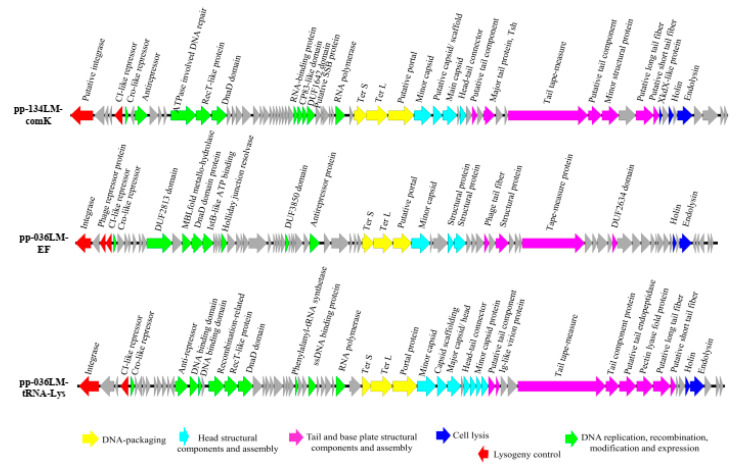
Genome annotation of prophage sequences present in the genomes of *L. monocytogenes* 134LM and 036LM.

The GC content of prophage pp-134LM-comK and pp-036LM-tRNA-Lys was 37.8%, while GC content of prophage pp-036LM-EF was 37.1%. Genome annotation of three prophages (Figure 2) revealed that prophage pp-134LM-comK contained 68 ORFs, of which 29 ORFs were assigned functions. Prophage pp-036LM-EF contained 67 ORFs, of which 32 ORFs were assigned functions. Prophage pp-036LM-tRNA-Lys contained 81 ORFs, of which 23 ORFs were assigned functions. Overall, the annotation of all three prophage sequences contained six main gene clusters: (i) lysogeny control; (ii) DNA replication, recombination, modification and gene expression; (iii) DNA packaging; (iv) head structural component and assembly; (v) tail and base plate; and (vi) cell lysis. In addition, the questionable prophage identified in both *L. monocytogenes* genomes, has been previously reported as a monocin [60,65]. These elements harbored a complete *lmaDCBA* operon identified as a virulence factor of *L. monocytogenes.*

### 3.3. Sequence Comparison of Prophages and Their Corresponding Induced Phages

**Figure 3:**
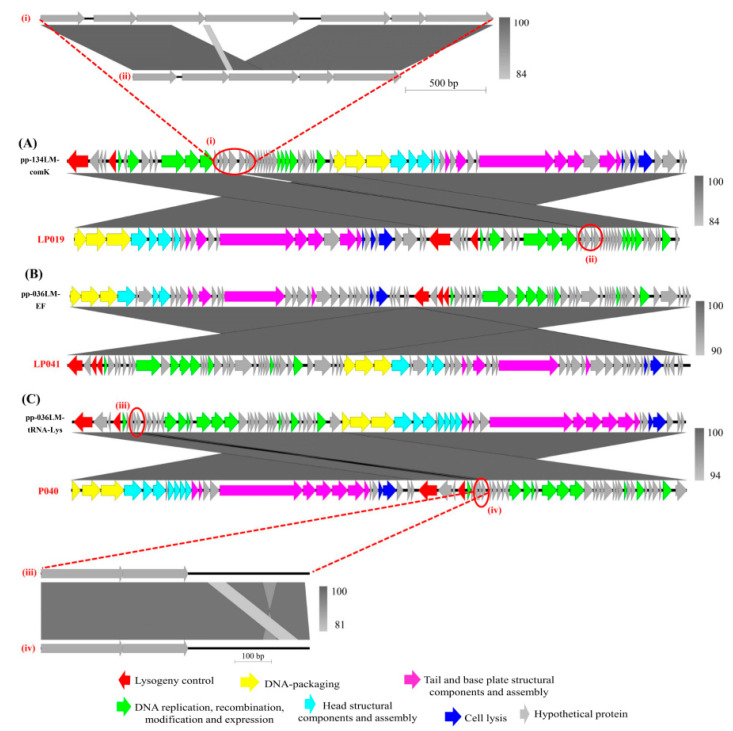
Genome comparison of the prophage sequences and their corresponding induced *Listeria* phages: (**A**) prophage pp-134LM-comK and phage LP019; (**B**) prophage pp-036LM-EF and phage LP041; (**C**) prophage pp-036LM-tRNA-Lys and phage LP040. The zoomed panel indicates variations in the sequences of prophage and the corresponding induced phage. The shade of grey between genomes indicates the level of nucleotide similarity (darker is higher similarity).

Overall, sequence comparison of prophages and the corresponding induced phages showed similar genes and gene functions (Figure 3). However, genome organization of the prophage was different from the induced phages. Specifically, the lysogeny control cluster and the induced phage early genes, which encoded for products of DNA replication, recombination, modification, and expression were located in the downstream of the prophage sequences. In contrast, the induced phage late genes encoding for products of DNA packaging, head, tail, and base plate structural and assembly proteins, were in the upstream of the prophage sequences. The phage integrase gene is always arranged in the downstream of prophage genome.

Several sequence variations were observed regions (labeled i to iv in Figure 3) of the prophage sequences. For example, an additional fragment of 1028 bp was present in the sequence of prophage pp-134LM-comK as compared to its corresponding induced phage LP019 sequence. This fragment encodes for hypothetical proteins ORF52–ORF55 of *Listeria* phage vB_LmoS_293. A 55-bp-fragment with some nucleotide modifications was found in the sequence of prophage pp-036LM-tRNA-Lys, thus differentiating this from the sequence of corresponding induced phage LP040. This could be a single/multiple nucleotide polymorphisms changed between the two sequences (induced phage and integrated prophage).

### 3.4. Prophages Inserted in Multiple Loci of L. monocytogenes Host Genomes

A total of 61 intact prophages detected by PHASTER within the genomes of 35 selected *L. monocytogenes* strains and an additional three prophages from two lysogens newly sequenced here were used for further analysis. Overall, these prophages were inserted into seven different insertion loci of *L. monocytogenes* genomes (Table 1). Prophages of multiple lysogenic strains showed common insertion sites, such as *comK* gene (n = 18), tRNA-Lys (n = 15) and tRNA-Arg (n = 14), followed by tRNA-Ser (n = 7), EF-Ts (n = 5), Ribosomal protein S9 (n = 3), and tRNA-Thr (n = 2).

The results reveal that many *L. monocytogenes* genomes with sequence similarity show a high degree of prophage conservation despite these strains being isolated from different sources, distinct geographical locations, and different times. For example, the strain J1776 (from human), J1926 (from food), and J1817 (from environment), shared 99.99–100% sequence similarity by ANIb (Supplement Table S1). They also harbored a conserved *comK*-prophage with 100% nucleotide sequence similarity. The *L. monocytogenes* strain L99 (from food, Netherlands, 1950), HCC23 (from animal, USA, 2000), and M7 (from human, China, 2009) shared 99.99–100% sequence similarity, and they contained the same set of prophages (including tRNA-Lys prophage with the size of 39,369–39,426 bp, tRNA-Arg prophage with the size of 41,757–41,788 bp and ribosomal protein S9 prophage with the size of 41,172–41,661 bp), with 85–100% nucleotide sequence similarity as determined by BLASTn. Among 12 strains, *L. monocytogenes* ST1 included for the analysis, *L. monocytogenes* lysogens 134LM (food source), and PNUSAL000474 (unknown source) shared 99.94% sequence similarity by ANIb; they also harbored a highly conserved *comK*-prophage with 90% nucleotide sequence similarity.

However, many *L. monocytogenes* genomes with high sequence similarity contained different prophages. In other words, the prophage sequence and their insertion sites also contribute to the genomic diversity of *L. monocytogenes* genomes. For instance, although the genome of the lysogenic isolate 134LM (from food, Thailand, 2013) showed high sequence similarity (99.97%) to the genomes of three *L. monocytogenes* strains 02-1792, 02-1289, and 02-1103 (from food/ human, Canada, 2002) as identified by ANIb, prophage pp-134LM-comK was found in a different cluster than prophages of the 3 strains 02-1792, 02-1289 and 02-1103 whose insertion sites were tRNA-Lys gene. Similarly, the *L. monocytogenes* 036LM (from environment, Thailand, 2013) and five *L. monocytogenes* strains J1-108, 81-0592, 81-0558, 81-0861 and 10-0809 (from food/human, Canada, 1981) shared 99.71% sequence similarity by ANIb. These strains contained a highly similar tRNA-Lys prophage by 99% nucleotide sequence similarity. The differences were from an extra 48,684 bp of the EF-TS-prophage inserted in the genome of the *L. monocytogenes* 036LM. Another example, an additional tRNA-Arg prophage, increased the distance of *L. monocytogenes* Scott A sequence (from human, USA, 1983) to three sequences of *L. monocytogenes* 02-1792, 02-1289, 02-1103 (from food/human, Canada, 2002).

### 3.5. Variation of Prophage Sequences Found in L. monocytogenes Genomes

**Figure 4:**
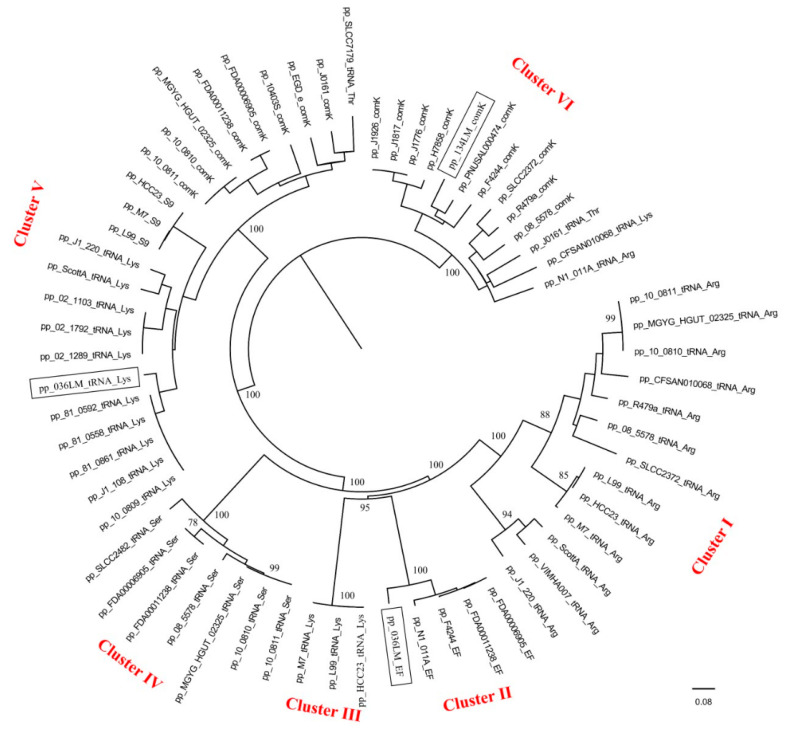
Phylogenetic tree based on full genome comparison of prophage sequences detected in *L. monocytogenes* genomes. Tree was constructed using whole genome-based phylogenetic analysis. GBDP pseudo-bootstrap support values from 100 replicates are given on the branches if support is ≥70%. The scale represents homology %. The three prophage sequences in the genomes of the two *L. monocytogenes* lysogens sequenced in this study are marked by a square.

Full genome comparison of 64 prophage sequences by VICTOR classified those into six distinct clusters, I to VI (Figure 4), supported by a bootstrap value of 100. Prophages with a single insertion site were often classified together. To specify, 13/14 prophages with the tRNA-Arg insertion site were classified together in cluster I. All five prophages with the EF-Ts insertion site were classified in cluster II. Seven prophages with the tRNA-Ser insertion site were classified in cluster IV. In contrast, only 3 of 15 prophages with the tRNA-Lys insertion site were classified in cluster III. However, prophages with various insertion sites were classified into clusters V and VI. Cluster V was the largest cluster, comprising 23 prophages with multiple insertion sites: *comK*, tRNA-Lys, tRNA-Thr, and ribosomal protein S9. In addition, cluster VI contained 13 prophages with four insertion sites: *comK*, tRNA-Lys, tRNA-Arg, and tRNA-Thr.

Linear genome visualizations of prophages were generated using Easyfig 2.1 (Supplement Figure S1). Result shows the overall relatedness of prophages despite various prophage insertion sites. For the clusters I to IV, which have prophages with a single insertion site, the integrase gene and nearby hypothetical proteins showed high similarity, while the genes, which encoded for DNA packaging shows higher diversity. In contrast, the two clusters (V and VI) that contained prophages with multiple insertion sites show no relatedness or very low similarity for the integrase gene and nearby hypothetical proteins. These prophages with different insertion sites were classified together because late genes show close relatedness.

Three prophages found in the two lysogens sequenced in this study were classified into clusters II, V, and VI. Interestingly, these prophage sequences were closely related to prophages of *L. monocytogenes* strains from various sources. For example, the pp-036LM-EF prophage showed high similarity to prophage of *L. monocytogenes* strains N1-011A, FDA00011238, and FDA00006905 (from environment, USA) and F4244 (from human, USA) by 96% nucleotide sequence similarity. The prophage pp-036LM-tRNA-Lys showed high similarity to prophage of *L. monocytogenes* strains J1-108, 81-0592, 81-0558, 81-0861, and 10-0809 (from food/human, Canada) by 99% nucleotide sequence similarity. The *comK*-prophage of 134LM was the most closely related to a *comK*-prophage of *L. monocytogenes* strains PNUSAL000474 (unknown source, USA) and F4244 (from human, USA) by 90% nucleotide sequence similarity.

### 3.6. Comparative Analysis of the Newly Sequenced Induced Phages and the Temperate Listeria Phages from Other Regions

**Figure 5:**
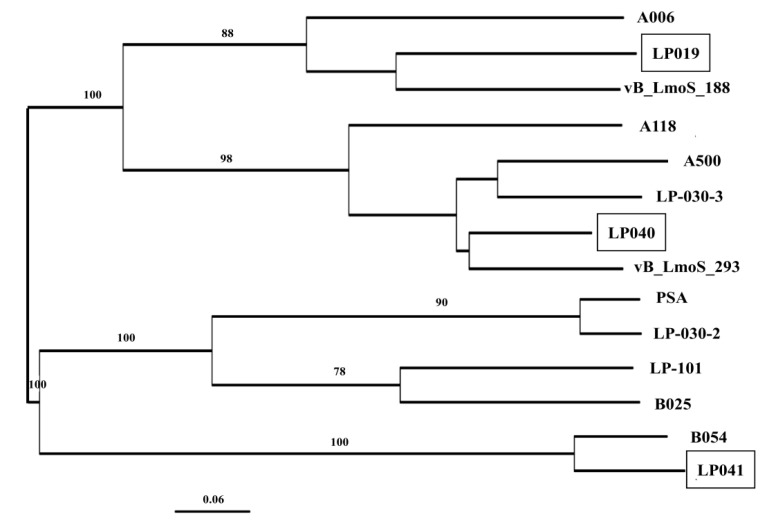
Phylogenetic tree of the newly sequenced phages (in a box) and previously sequenced temperate phages. Tree was constructed using the whole genome-based phylogenetic analysis. GBDP pseudo-bootstrap support values from 100 replicates are given on the branches if support is ≥70%.

Genomes of phages sequenced here were closely related to several temperate phages (Figure 5). Phage LP019 was closely related to *Listeria* phage vB_LmoS_188, which was a temperate phage previously isolated from wild mushroom in Ireland. Phage LP040 was closely related to phage vB_LmoS_293, which was also a temperate phage previously isolated from mushroom compost in Ireland. Phage LP041 was closely related to a unique *Listeria* phage B054, which was previously induced from *L. innocua* WSLC 2054 in the U.S.

**Figure 6:**
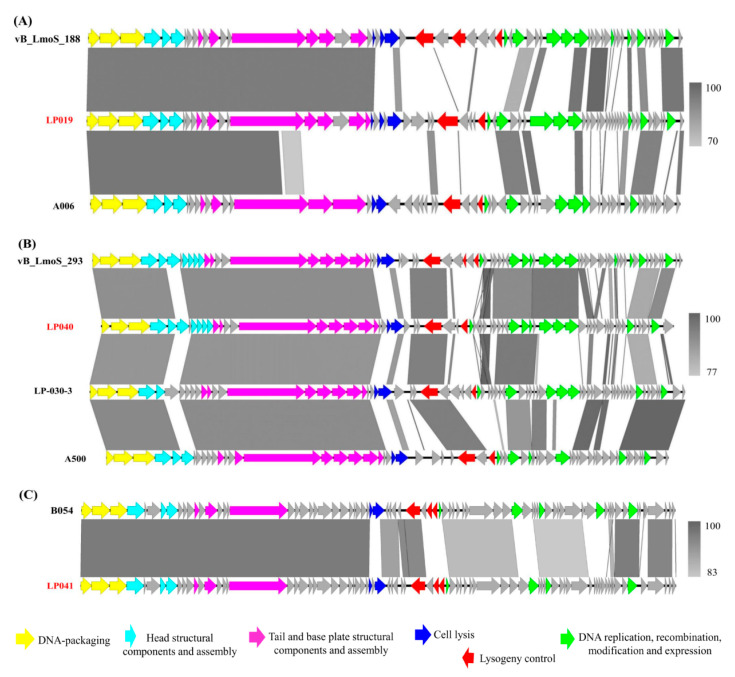
Genome comparison of the newly sequenced phages (performed in previous study [22]) and closely related temperate phages. (**A**) LP019, (**B**) LP040, and (**C**) LP041; predicted genes were indicated by arrows and colored according to major gene functions. The shade of gray drawn between genomes represented the level of nucleotide similarity.

Genome sequence comparison of each newly sequenced *Listeria* phage and the closely related phages indicates some regions with high nucleotide sequence similarity (>90%), especially the late genes, which encoded for DNA packaging, head, tail, and base plate structure (Figure 6). Overall, partial regions of the lysogeny control cluster and early genes encoded for DNA replication, recombination, modification, and gene expression presented low similarity between each newly sequenced phage, and the closely related phage. However, the integrase gene and nearby hypothetical proteins of phage LP040 and phage LP041 showed high similarity to those of phage vB_LmoS_293 and phage B054, respectively. Interestingly, the Cro/CI repressor and anti-repressor genes involving in the phage genetic switch were found to be less conserved in all the phages analyzed. Sequence comparison of the induced phage LP019 and isolated temperate phage vB_LmoS_188 showed low similarity in most regions, except the late genes. Overall, genomes of our induced phages from seafood and a seafood-related environment were closely related to the temperate phages from natural environments (i.e., wild mushroom, mushroom compost, and dairy farm) or induced phages from lysogens of other *Listeria* species.

## 4. Discussion

### 4.1. Prophages and Prophage-Related Regions Contribute to the Diversity of L. monocytogenes Genomes

Overall, the genomes of these lysogens displayed typical genome features of *L. monocytogenes* such as assembly size (2.8–3 Mb), GC content (~38%) as reported previously [13,66]. However, the number of unique rRNA operons (16S-23S-5S) found in the genome of 134LM and 036LM were lower than the number of rRNAs of *Listeria* genomes as commonly reported, which typically showed 13–18 repeat rRNA regions across the chromosome [13,64]. This could be due to the fact the ‘repeat’ rRNA regions would all map to the same gene when using short read sequencing. Another possible reason for the missing rRNAs is that they could be present in gaps between contigs of the two *L. monocytogenes* lysogens sequence genomes [44].

The two genomes of lysogenic *L. monocytogenes* newly sequenced here shared 99.94% sequence similarity by ANIb. The two intact prophages in the lysogenic genome of 036LM could account for the difference, compared to the genome of 134LM. In addition, genome sequences of both lysogens showed high similarity to the genome of *L. monocytogenes* reference strain F2365, but prophage was not detected in F2365 and no insertion site was found. Furthermore, the extra EF-Ts prophage in 036LM or the tRNA-Arg prophage in *L. monocytogenes* Scott A made the difference between these strains and their closely related strains. These findings suggest that prophage sequences could contribute to evolutionary changes of *L. monocytogenes* genomes. Similarly, Orsi et al. (2008) reported that the differences in a 42-kb-prophage sequence differentiate four similar genome backbone *L. monocytogenes* isolates of a strain which persisted over 12 years, indicating the importance of prophages in differentiation of closely related *L. monocytogenes* [12].

Prophages were found in different loci of *L. monocytogenes* genomes as indicated by various insertion sites detected. The *comK* gene and tRNA-Lys gene were reported as the most common insertion site for *Listeria* prophages. Previous studies have shown that multiple *L. monocytogenes* strains such as J0161, J2818, R479a, HCC23 and J1-108 containing *comK* and/or tRNA-Lys prophage(s) [9,13]. Lysogens containing *comK*-prophages have been previously reported to facilitate the survival over 10 years in foods and food processing environments [11]. The *comK*-prophage could also help the *Listeria* lysogen escape from activated macrophages, facilitating the virulence of *L. monocytogenes* when this bacteria infects a human host [67]. This prophage insertion site could therefore enhance the survival and virulence of *Listeria* lysogen. In addition, the EF gene was another insertion site for *Listeria* prophages detected in this study. This is considered as a rare insertion site, which has also previously been found in a few strains of *L. monocytogenes* such as SLCC2755 [13] and *L. innocua* strain Clip11262 [59]. The EF gene plays an important role in facilitating the translational elongation of protein synthesis of the *L. monocytogenes*. Overall, prophage insertion loci could be influenced by the integrase gene or the bacterial attachment sites [68].

Full genome comparison showed high sequence similarity by ANIb analysis between *L. monocytogenes* 134LM newly sequenced here and *L. monocytogenes* strains on the database such as 02-1792, 02-1289, and 02-1103. However, the prophage in the genome of *L. monocytogenes* 134LM showed a different insertion site and was found in a different phylogenetic cluster than those closely related strains. These findings suggest that prophage sequences and the insertion sites could provide additional information for identifying the variations in the genomes of *Listeria* beyond the analysis by ANIb. Similarly, previous studies have revealed that the genetic diversity of *L. monocytogenes* was affected by gain or loss of genetic islands, especially the presence of prophage sequences [9,13,69]. In addition, *L. monocytogenes* strains showing prophage with the same genome size), insertion site, and classified into the same cluster, could have relatedness confirmed by ANIb analysis. Therefore, data from the detection of prophages, differentiation of insertion sites in specific loci of *Listeria,* and prophage sequence comparison, together with the ANIb confirmation, could be used to further classify *L. monocytogenes* strains.

We found that the prophage insertion sites could be diverse in *L. monocytogenes* genomes, which contributes to the genomic diversity of *L. monocytogenes* genomes. Moreover, variation of prophage sequences was found in the genomes of *L. monocytogenes*, suggesting diverse types of prophages were found in *L. monocytogenes* genomes, which play an important role in the host genome diversity. Previous studies have reported the role of prophages, as a contributor to the *Listeria* diversity [56]. Diverse types of prophages found in the genome of *Listeria* lysogens could contribute to the variation of host genomes as mentioned in a previous study [9,25].

### 4.2. Change in the Genome Organization between Prophage and the Corresponding Induced Listeria Phage

Prophages showed genome features with typical six gene clusters of temperate *Listeria* phages [17,18,19,20]. However, slight differences in the genome organization of prophage and the corresponding induced phage were observed. This observation could be explained by the phage packaging strategy. Induced phages LP019, LP040, and LP041 were predicted as circularly permuted, terminally redundant dsDNA phages [22], suggesting the opportunity for the phage genome to recombine both arms within the terminally redundant ends. As a result, the genome of each induced phage could become a circular dsDNA molecule before integrating into the host chromosome at an attachment site and becoming a prophage [11,18]. Furthermore, the additional sequence in prophage pp-134LM-comK or the altered nucleotide sequence in prophage pp-036LM-tRNA-Lys led to variations between prophage and the corresponding induced phage. This could be due to the gaining of accessory genes by either horizontal gene transfer by phage transduction [70,71] or rapid niche-specific adaptation as proposed by Verghese et al. (2011) [11]. These findings are consistent with a previous report that alterations of *Listeria* genome during prophage induction or the acquisition of prophage genes is a main source of *L. monocytogenes* genome diversity [13].

### 4.3. Uniqueness of Induced Phages and Temperate Listeria Phages from Various Sources and Regions

Genomes of the induced phages from seafood and a seafood-related environment are closely related to the temperate phages from natural environments (specifically, wild mushroom, mushroom compost, and dairy farm) or induced phages from lysogens of other *Listeria* species. However, low sequence similarity was observed in the region of cell lysis and lysogeny control between the induced phages and the closely related temperate phages. In the cell lysis cluster, neither holin nor endolysin genes were conserved among the examined phages in this study. Similar to report of previous study by Dorscht et al. (2009), genes encoding for holin and endolysin showed a low similarity among the *Listeria* phages included in the analysis. These temperate and induced phages were not only from different sources, but also from distinct regions in the world, e.g., Thailand, Ireland, and the U.S.

The lysogeny control cluster of phages LP040 and LP041 showed high percentage (%) similarity to their closely related temperate phages. This suggests the possibility that this phage may insert into the same genomic locus or have the same phage insertion site. This could be from the phage attachment site and the bacterial attachment site recombining between two DNA recognition sequences, resulting in the phage genome integrated into the bacterial chromosome by site-specific recombination [69,72]. This hypothesis was confirmed when we found that the induced phage LP041 and its closely related phage B054 was excised from the 3′ end of translation elongation factor gene, whereas the LP040 and the closely related phage A500 integrated into the tRNA-Lys [19]. However, the Cro/CI repressor and anti-repressor genes involving in the phage genetic switch, which is responsible for expression of other phage genes leading to the launch of the lytic pathway or maintaining the lysogenic cycle [73], were found to be less conserved in all of the phages analyzed. These findings suggest a different potential mechanism required for prophage induction from the bacterial chromosome.

## 5. Conclusions

Here, we reported the draft genome sequences of two lysogenic isolates of *L. monocytogenes* (134LM and 036LM) obtained from seafood and a seafood-related environment. Intact prophages were found in the genomes of these two lysogens. A comparative prophage sequence analysis suggests that various prophage insertion sites, as well as diverse prophage sequences, could contribute to the diversity of *Listeria* in food processing environments. Overall, the study here provides a model for comparative analysis among prophages in diverse genomes of *L. monocytogenes* lysogens. Data from the detection of prophages, differentiation of insertion sites in specific loci of *Listeria,* and prophage sequence comparison together with the ANIb analysis, could further classify closely related *L. monocytogenes* strains/isolates. This study also suggests the potential development for using a prophage as a typing tool for monitoring and surveillance of foodborne pathogen contamination and transmission.

## Figures and Tables

**Table 1:** *L. monocytogenes* genome sequences presenting prophages and prophage-related regions.

Information of the Lysogen									Length (bp) of Prophage Identified in Each Insertion Site							Reference **
Strain/Isolates ID	Sequence Type	Serogroup	Lineage	CC	Source	Year	Country	Accession No.	*comK*	tRNA-Lys	tRNA-Arg	tRNA-Ser	tRNA-Thr	EF-Ts	Ribosomal Protein S9	
**134LM**	**ST1**	IVb *	**I**	**CC1**	**Food**	**2013**	**Thailand**	**QSWG00000000.1**	**39,532**							This study ***
PNUSAL000474	ST1	IVb *	I	CC1	Unknown	-	USA	JSWS01000001.1	38,978							Unpublished
J1-108	ST1	4b	I	CC1	Food	1981	Canada	CP006596.1		39,322						[49]
81-0558	ST1	4b	I	CC1	Human	1981	Canada	CP007525.1		39,322						[50]
81-0861	ST1	4b	I	CC1	Human	1981	Canada	CP006874.1		39,322						[51]
81-0592	ST1	4b	I	CC1	Human	1981	Canada	CP007526.1		39,322						[52]
10-0809	ST1	4b	I	CC1	Human	1981	Canada	CP007167.1		39,322						[50]
02-1792	ST1	4b	I	CC1	Food	2002	Canada	CP007461.1		41,248						[50]
02-1289	ST1	4b	I	CC1	Human	2002	Canada	CP007460.1		41,248						[50]
02-1103	ST1	4b	I	CC1	Human	2002	Canada	CP007459.1		41,248						[50]
Scott A	ST1	4b	I	CC2	Human	1983	USA	CM001159.1		42,003	37,502					[53]
**036LM**	**ST1**	IVb *	**I**	**CC1**	**Environment**	**2013**	**Thailand**	**QOUY00000000.1**		**39,488**				**48,684**		**This study**
VIMHA007	ST2	4b	I	CC2	Human	-	Russia	CP018149.1			36,576					Unpublished
J1-220	ST2	4b	I	CC2	Human	1979	USA	CP006046.3		42,348	38,700					[49]
SLCC2482	ST3	7	I	CC3	Human	1966	-	FR720325				33,409				[13]
N1-001A	ST3	IIb *	I	CC3	Environment	-	USA	CP006597.1			38,108			47,525		Unpublished
CFSAN010068	ST5	IIb *	I	CC5	Food	2014	USA	CP014250.1		41,864	44,935					[54]
10-0810	ST5	1/2b	I	CC5	Human	1996	Canada	CP007168.1	41,583		45,827	33,225				[55]
10-0811	ST5	1/2b	I	CC5	Human	1996	Canada	CP007169.1	41,583		45,827	33,225				[55]
MGYG-HGUT-02325	ST5	IIb *	I	CC5	Human	-	UK	LR698978.1	41,583		45,827	33,225				Unpublished
FDA00006905	ST5	IIb *	I	CC5	Environment	2011	USA	CP023052.1	40,883			32,205		47,272		Unpublished
FDA00011238	ST5	IIb *	I	CC5	Environment	2017	USA	CP023050.1	40,883			32,203		47,272		Unpublished
J1776	ST6	4b	I	CC6	Human	2002	USA	CP006598	39,825							[56]
J1926	ST6	4b	I	CC6	Food	2002	USA	CP006600	39,825							[57]
J1817	ST6	4b	I	CC6	Environment	2002	USA	CP006599	39,810							[56]
H7858	ST6	4b	I	CC6	Food	1998	USA	AADR00000000	40,484							[14]
R479a	ST8	1/2a	II	CC8	Food	1996	Denmark	HG813247.1	38,927		42,048					[58]
J0161	ST11	1/2a	II	CC11	Human	2000	USA	NC_017545.1	41,530				38,519			[12]
EGD-e	ST35	1/2a	II	CC9	Animal	1926	UK	NC_003210.1	41,459							[59]
10403S	ST85	1/2a	II	CC7	Human	1968	USA	CP002002	37,474							[9]
SLCC7179	ST91	3a	II	CC14	Food	1986	Austria	FR733650					38,696			[13]
SLCC2372	ST122	1/2c	II	CC9	Human	1935	UK	FR733648	38,319		42,317					[13]
L99	ST201	4a	III	CC69	Food	1950	Netherlands	FM211688.1		39,372	41,757				41,172	[60]
HCC23	ST201	4a	III	CC69	Animal	2000	USA	CP001175.1		39,369	41,757				41,661	[61]
M7	ST201	4a	III	CC69	Human	2009	China	NC_017537		39,426	43,788				41,172	[62]
F4244	ST1347	4b	I	CC6	Human	1991	USA	CP015508.1	40,067					47,934		[63]
08-5578	ST292	1/2a	II	CC8	Human	2008	Canada	CP001602	39,425		44,365	36,088				[64]

* Serotype group ‘serogroup’ was determined by comparison to MLST [35]; ** Unpublished: the *L. monocytogenes* genome sequences available in the NCBI database, but not yet reported in any publication. *** *L. monocytogenes* lysogens sequenced in this study are bolded.

**Table 2:** Temperate *Listeria* phage genome sequences used for comparative analysis.

Temperate Listeria Phage	Genome Size (bp)	NCBI Accession No.	Reference
LP019	38,601	MH341451	[22]
LP040	39,585	MH341452	[22]
LP041	48,286	MH341453	[22]
A118	40,834	NC_003216.1	[18]
A006	38,124	NC_009815.1	[19]
B025	42,653	NC_009812.1	[19]
B054	48,172	NC_009813.1	[19]
A500	38,867	NC_009810.1	[19]
PSA	37,618	AJ312240.2	[17]
LP-101	43,767	NC_024387.1	[20]
LP-030-2	38,275	JX120799.2	[20]
LP-030-3	41,156	NC_024384.1	[20]
vB_LmoS_188	38,392	KP399677	[21]
vB_LmoS_293	40,759	KP399678	[21]

**Table 3:** Lysogenic *L. monocytogenes* draft genome information.

Description	PSU-KV-134LM (134LM)	PSU-KV-036LM (036LM)
No. of reads (pre-filtered)	27,525,582	24,257,104
No. of reads (post filtered)	21,137,912	19,286,526
Assembly size (bp)	2,953,877	3,000,399
Sequencing coverage	715×	643×
GC content (%)	37.80	37.83
Number of contigs	11	11
Shortest contig size (bp)	1778	3570
Longest contig size (bp)	766,006	1,291,185
N50 value	476,941	556,523
No. of predicted CDSs	2955	3027
No. of unique rRNAs (5S, 16S, 23S)	2, 1, 1	1, 1, 1
No. of tRNAs	54	52
No. of prophage regions	2	3
Length of intact prophage (bp)	39,532	48,684 and 39,488
Length of questionable prophage (bp)	10,729	10,729

## Supplementary Materials

The following are available online at https://www.mdpi.com/article/10.3390/microorganisms9071354/s1, Table S1: ANIb values of 2 *L. monocytogenes* lysogens sequenced in this study and 35 *L. monocytogenes* strains/ isolates sequenced previously and Figure S1: Linear genome visualization of the prophage sequences in each of the six identified clusters conducted using Easyfig 2.1.
