# Supplementary material for: Genomic Analysis of Prophages Recovered from Listeria monocytogenes Lysogens Found in Seafood and Seafood-Related Environment
Source: Microorganisms. 2021 Jun 22;9(7):1354. doi: 10.3390/microorganisms9071354 (PMC8303350; doi:10.3390/microorganisms9071354)
Supplement: Supplementary file 1 [file microorganisms-09-01354-s001.zip › microorganisms-1195800-supplementary.pdf]

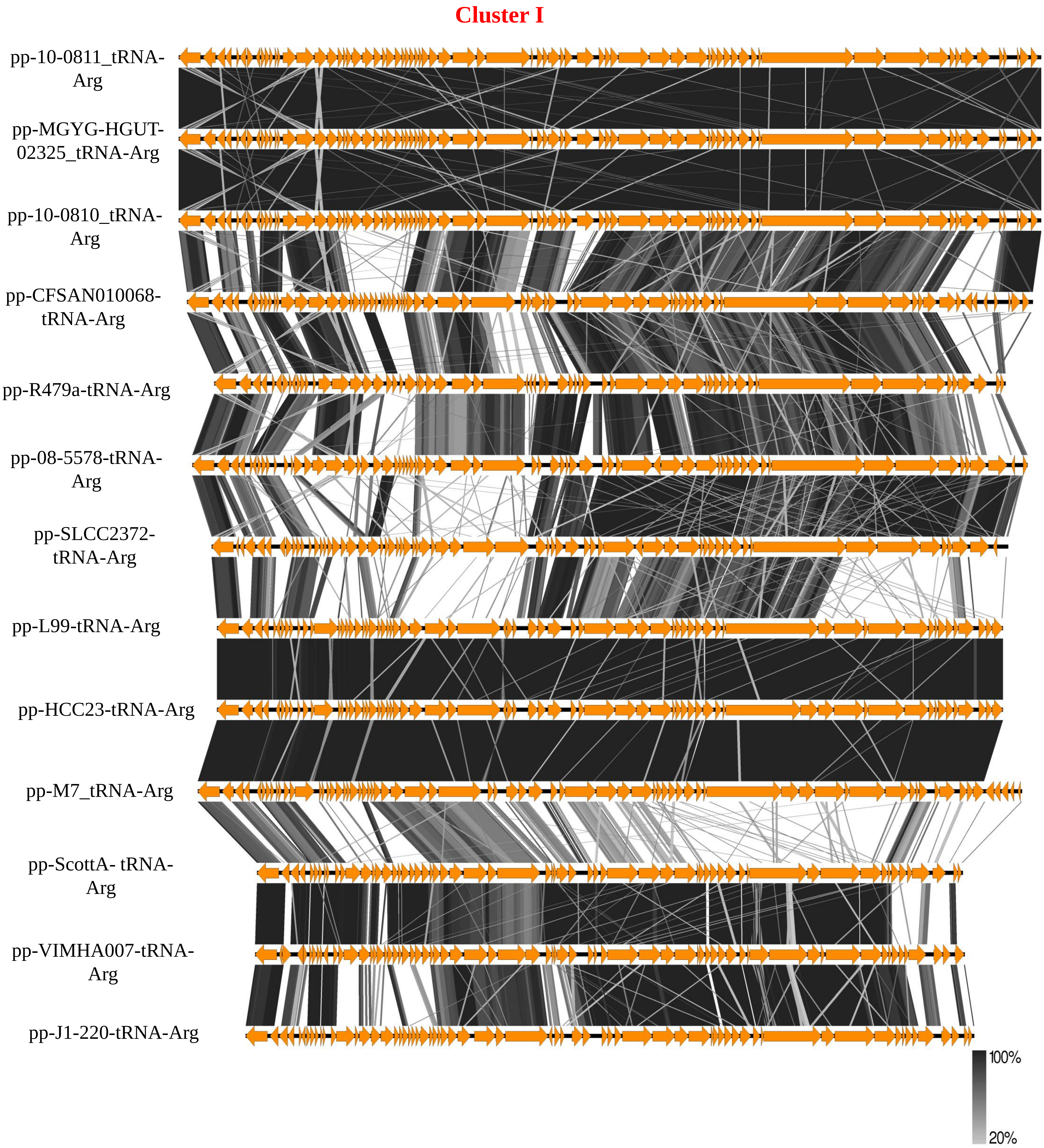
Cluster I
pp-10-0811_tRNA-Arg
pp-MGYG-HGUT-02325_tRNA-Arg
pp-10-0810_tRNA-Arg
pp-CFSAN010068-tRNA-Arg
pp-R479a-tRNA-Arg
pp-08-5578-tRNA-Arg
pp-SLCC2372-tRNA-Arg
pp-L99-tRNA-Arg
pp-HCC23-tRNA-Arg
pp-M7_tRNA-Arg
pp-ScottA- tRNA-Arg
pp-VIMHA007-tRNA-Arg
pp-J1-220-tRNA-Arg
100%
20%

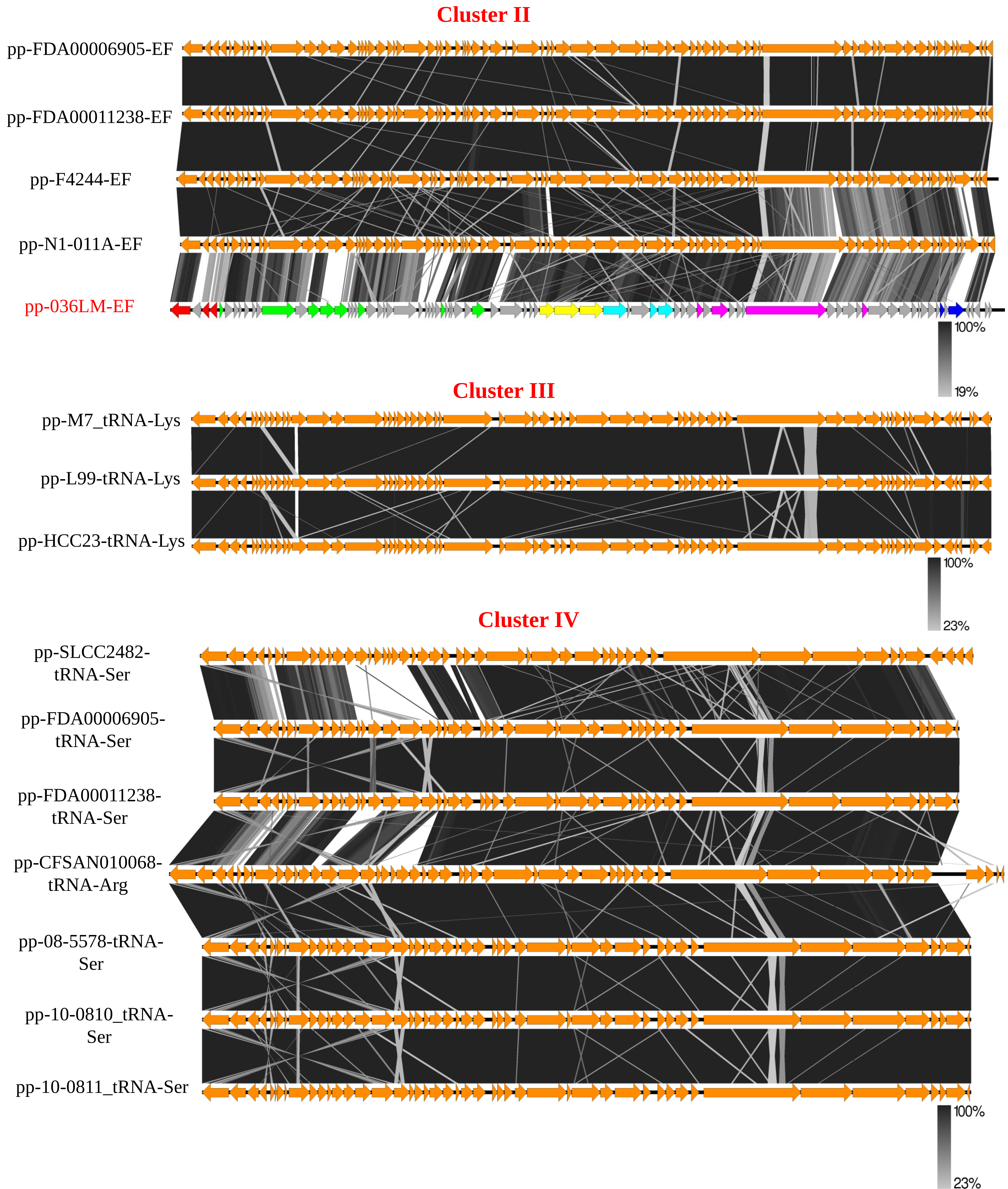

Cluster II
pp-FDA00006905-EF
pp-FDA00011238-EF
pp-F4244-EF
pp-N1-011A-EF
pp-036LM-EF
100%
19%
Cluster III
pp-M7_tRNA-Lys
pp-L99-tRNA-Lys
pp-HCC23-tRNA-Lys
100%
23%
Cluster IV
pp-SLCC2482-tRNA-Ser
pp-FDA00006905-tRNA-Ser
pp-FDA00011238-tRNA-Ser
pp-CFSAN010068-tRNA-Arg
pp-08-5578-tRNA-Ser
pp-10-0810_tRNA-Ser
pp-10-0811_tRNA-Ser
100%
23%

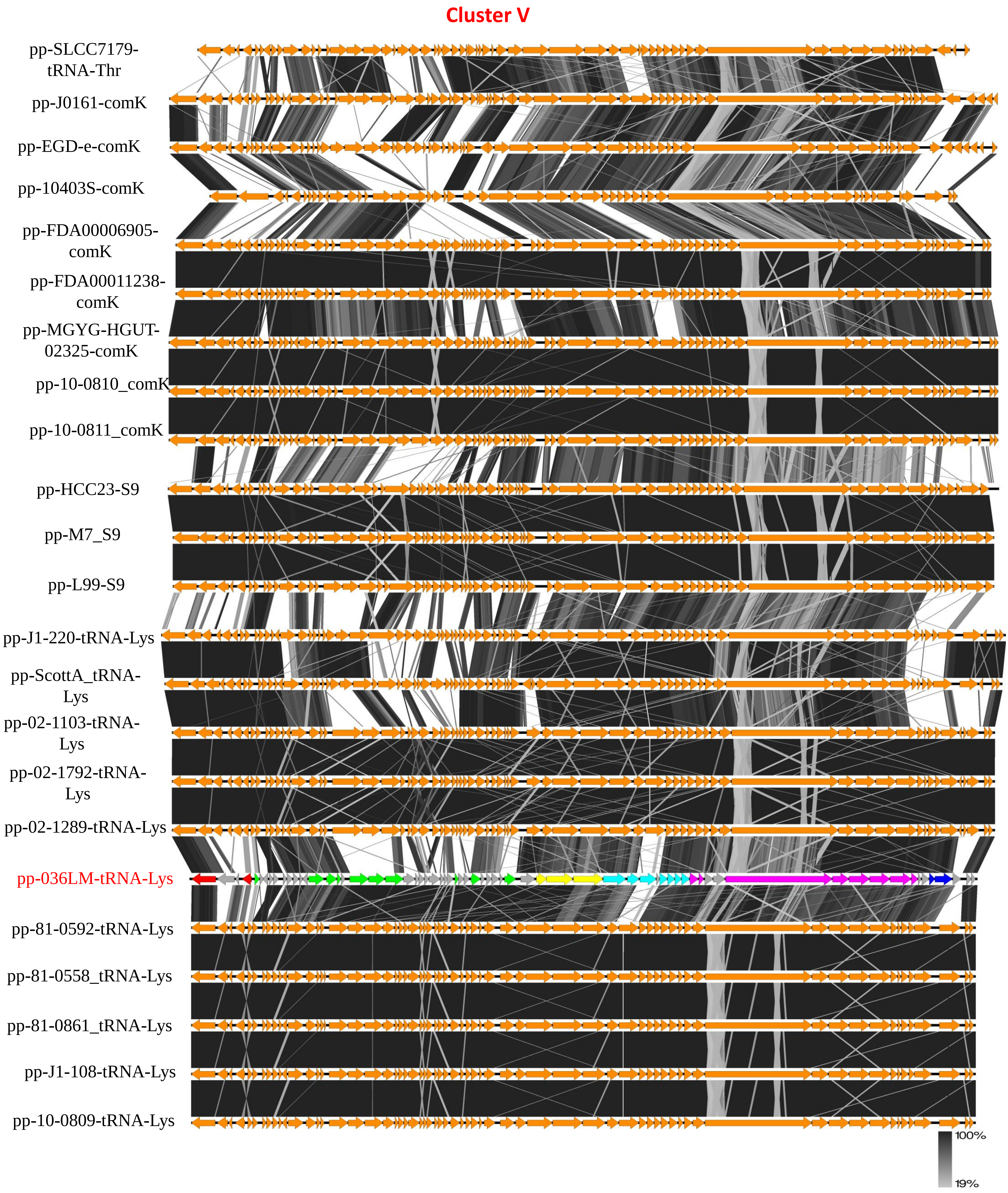

Cluster V
pp-SLCC7179-tRNA-Thr
pp-J0161-comK
pp-EGD-e-comK
pp-10403S-comK
pp-FDA00006905-comK
pp-FDA00011238-comK
pp-MGYG-HGUT-02325-comK
pp-10-0810_comK
pp-10-0811_comK
pp-HCC23-S9
pp-M7_S9
pp-L99-S9
pp-J1-220-tRNA-Lys
pp-ScottA_tRNA-Lys
pp-02-1103-tRNA-Lys
pp-02-1792-tRNA-Lys
pp-02-1289-tRNA-Lys
pp-036LM-tRNA-Lys
pp-81-0592-tRNA-Lys
pp-81-0558_tRNA-Lys
pp-81-0861_tRNA-Lys
pp-J1-108-tRNA-Lys
pp-10-0809-tRNA-Lys
100%
19%

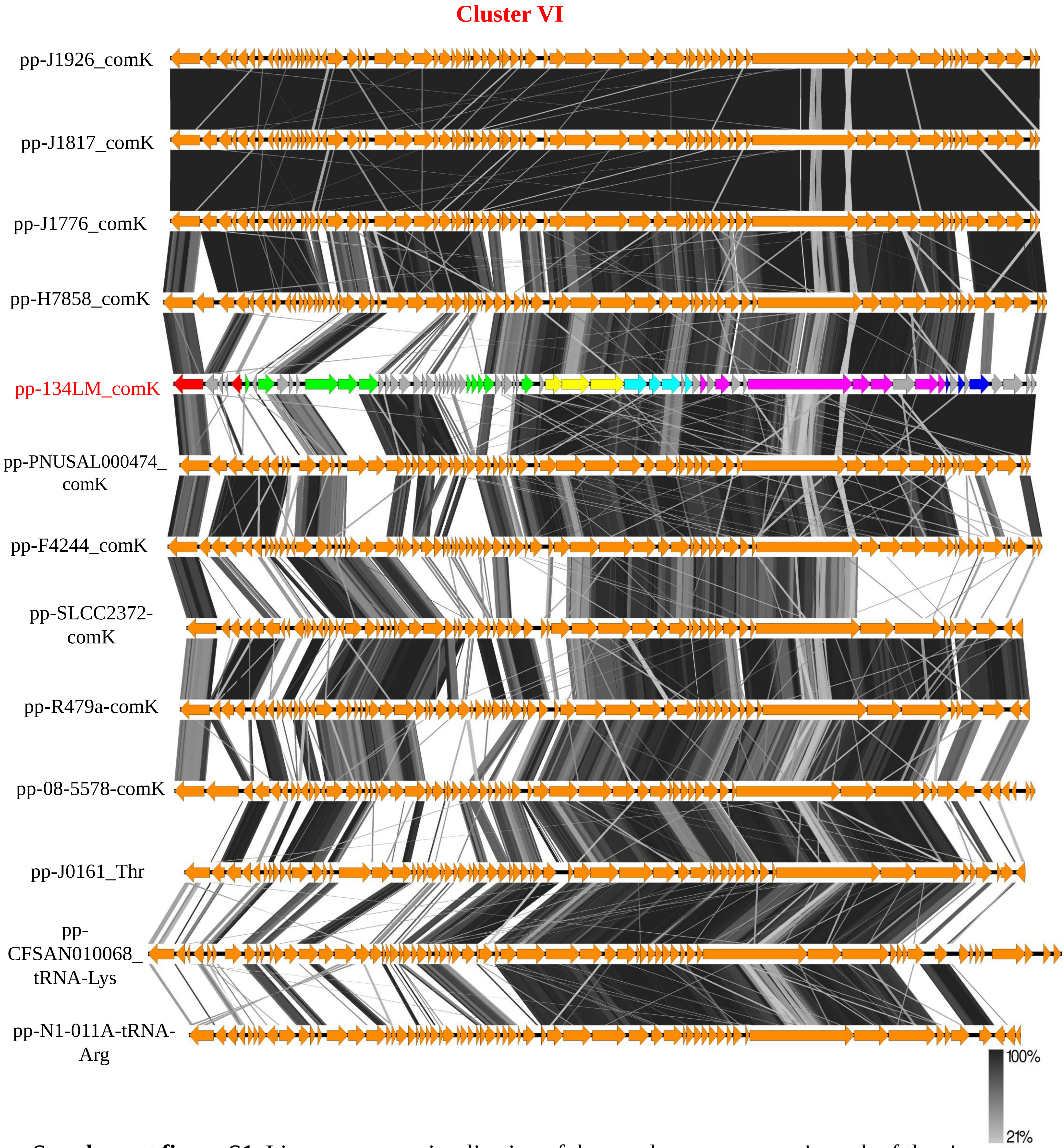

Supplement figure S1. Linear genome visualization of the prophage sequences in each of the six identified clusters conducted using Easyfig 2.1. The shade of grey between genomes indicates the level of nucleotide similarity (the darker, the higher similar).
